# P-1364. Cefiderocol Activity against *Pseudomonas aeruginosa* Clinical Isolates Carrying Metallo-β-lactamase Genes in United States and European Hospitals (2020–2023)

**DOI:** 10.1093/ofid/ofae631.1541

**Published:** 2025-01-29

**Authors:** Rodrigo E Mendes, Danielle Beekman, Maura Karr, Hank Kimbrough, Cory Hubler, Helio S Sader, Mariana Castanheira

**Affiliations:** JMI Laboratories, North Liberty, Iowa; Element Materials Technology/Jones Microbiology Institute, North Liberty, Iowa; Element Materials Technology/Jones Microbiology Institute, North Liberty, Iowa; Element Materials Technology/Jones Microbiology Institute, North Liberty, Iowa; Element Materials Technology/Jones Microbiology Institute, North Liberty, Iowa; JMI Laboratories, North Liberty, Iowa; JMI Laboratories, North Liberty, Iowa

## Abstract

**Background:**

Cefiderocol (FDC) is a siderophore cephalosporin that uses the iron transport systems of Gram-negative bacteria to optimize cell entry. FDC is stable to hydrolysis by serine and metallo-β-lactamases (MBL). FDC and comparator activities were analyzed against *P. aeruginosa* (PSA) carrying MBL genes, as part of the SENTRY Antimicrobial Surveillance Program.

**Figure:**
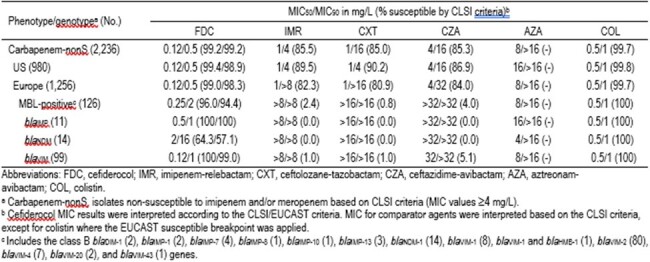

**Methods:**

9,573 PSA were collected from 36 sites in the US and 43 sites in Europe (EU) in 2020–2023. Susceptibility (S) testing used broth microdilution with cation-adjusted Mueller-Hinton broth (CAMHB) for comparators and iron-depleted CAMHB for FDC. CLSI/EUCAST breakpoints were used for FDC; whereas CLSI were applied for comparators (EUCAST for colistin [COL]). Isolates with MIC ≥4 mg/L (nonS by CLSI) for imipenem or meropenem were screened for β-lactamase genes.

**Results:**

Carbapenem-nonS PSA comprised 23.4% (2,236/9,573) of isolates, with 22.3% (980/4,400) and 24.3% (1,256/5,173) originating from US and EU sites, respectively (Table). 5.5% (124/2,236) of PSA carried MBL, and these PSA were mostly from EU (94.4%). FDC (98.3–99.4%S) had MIC_50_ of 0.12 mg/L and MIC_90_ of 0.5 mg/L against carbapenem-nonS PSA from the US and EU, whereas other agents had lower S (80.9–90.2%), except for COL (99.7–99.8%S). Aztreonam-avibactam inhibited only 48.4% and 57.6% of carbapenem-nonS PSA from the US and EU at MIC of ≤8 mg/L, respectively. FDC (MIC_50/90_, 0.25/2 mg/L; 94.4–96.0%S) had activity against the MBL-carrying PSA subset; in contrast, other agents had off-scale MIC_90_ (i.e. >8 mg/L), except for COL (100%S). All PSA carrying *bla*_IMP_ and *bla*_VIM_ were susceptible to FDC (MIC_90/100_, 1/2 mg/L), whereas higher FDC MIC (MIC_50/90_, 2/16 mg/L; 57.1–64.3%S) were noted against *bla*_NDM_-carrying PSA.

**Conclusion:**

FDC showed potent activity against carbapenem-nonS PSA clinical isolates from US and EU hospitals, including isolates carrying MBL genes, whereas newly launched BL/BLI didn’t show activity. FDC should be considered as an important option for the treatment of infections caused by these resistant subsets for which antibiotic treatment option are limited.

**Disclosures:**

**Rodrigo E. Mendes, PhD**, GSK: Grant/Research Support

